# A simple method for decellularizing a cell-derived matrix for bone cell cultivation and differentiation

**DOI:** 10.1007/s10856-021-06601-y

**Published:** 2021-09-15

**Authors:** Weidong Weng, Filippo Zanetti, David Bovard, Bianca Braun, Sabrina Ehnert, Tatiana Uynuk-Ool, Tina Histing, Julia Hoeng, Andreas K. Nussler, Romina H. Aspera-Werz

**Affiliations:** 1 Department of Trauma and Reconstructive Surgery, BG Trauma Center Tübingen, Siegfried Weller Institute for Trauma Research, Eberhard Karls University Tübingen, 72076 Tübingen, Germany; 2 PMI R&D, Philip Morris Products S.A., Quai Jeanrenaud 5, CH-2000 Neuchâtel, Switzerland

## Abstract

The extracellular matrix regulates cell survival, proliferation, and differentiation. In vitro two-dimensional cell experiments are typically performed on a plastic plate or a substrate of a single extracellular matrix constituent such as collagen or calcium phosphate. As these approaches do not include extracellular matrix proteins or growth factors, they fail to mimic a complex cell microenvironment. The cell-derived matrix is an alternative platform for better representing the in vivo microenvironment in vitro. Standard decellularization of a cell-derived matrix is achieved by combining chemical and physical methods. In this study, we compared the decellularization efficacy of several methods: ammonium hydroxide, sodium dodecyl sulfate (SDS), or Triton X-100 with cold or heat treatment on a matrix of Saos-2 cells. We found that the protocols containing SDS were cytotoxic during recellularization. Heat treatment at 47 °C was not cytotoxic, removed cellular constituents, inactivated alkaline phosphatase activity, and maintained the levels of calcium deposition. Subsequently, we investigated the differentiation efficiency of a direct bone coculture system in the established decellularized Saos-2 matrix, an inorganic matrix of calcium phosphate, and a plastic plate as a control. We found that the decellularized Saos-2 cell matrix obtained by heat treatment at 47 °C enhanced osteoclast differentiation and matrix mineralization better than the inorganic matrix and the control. This simple and low-cost method allows us to create a Saos-2 decellularized matrix that can be used as an in vivo-like support for the growth and differentiation of bone cells.

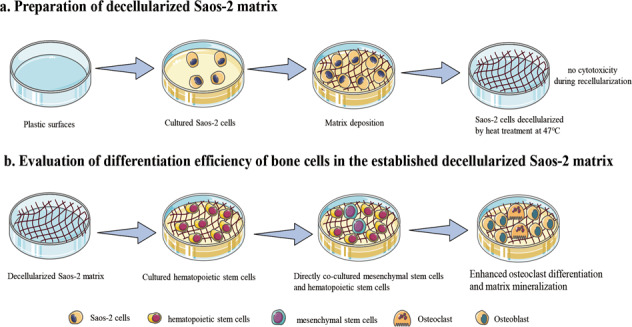

## Introduction

The extracellular matrix (ECM) is a network of proteins and carbohydrates [1] that provides structural support to cells and regulates cell migration, proliferation, and differentiation [1–3]. The composition of the ECM varies by tissue and organ [4]. Bone ECM is mainly composed of type I collagen embedded in mineral crystals (calcium phosphate [CaP] and hydroxyapatite) [5]. Type I collagen induces osteoblast and osteoclast differentiation by binding integrins of mesenchymal stem cells [6]. Several ECM proteins (α5 and β5 integrin chains) secreted by mesenchymal stem cells modulate the differentiation of these cells into osteoblasts [7]. Two ECM molecules, osteopontin and bone sialoprotein, when combined with αvβ3 integrin, can trigger osteoclast migration and adhesion to bone ECM as well as differentiation of preosteoclasts into mature osteoclasts [8]. Physiological mimicking of active bone formation and degradation processes occurring in vivo requires a feasible and reliable platform for bone cell cultivation and differentiation.

Decellularization is a promising method for removing cellular elements and maintaining ECM structure and functional constituents left by the cells on the substrate [9]. Tissues and organs are ideal sources of decellularized matrices; but the scarcity of native ECM sources restricts their large-scale application in in vitro experiments [10]. Inorganic matrices have been used as an alternative; the inorganic constituents of native bone ECM are hydroxyapatite and CaP, which are easy to obtain. Hydroxyapatite coatings improve surface bioactivity, but may not help cells maintain long-term stability [11]. CaP coatings exhibit excellent biocompatibility and osteoconductive features [12–14], and several studies have shown that CaP coatings promote differentiation of osteoclastic precursors to osteoclasts in favor of bone resorption [12, 13]. CaP coatings can also steer bone marrow stromal cells towards osteogenic differentiation [14]. Despite their apparent benefits, these inorganic matrices do not contain proteins or growth factors (e.g., transforming growth factor β, bone morphogenetic proteins, or collagen) that regulate bone cell differentiation and cellular metabolism during the development and remodeling of bone tissue. Regulation of cell behavior can be achieved by incorporating collagen, a major organic constituent of native bone ECM [15]. For instance, only the presence of both organic and inorganic components in native bone ECM facilitates annexin A8 expression, which is required for initial osteoclast cell fusion [15]. Therefore, a reliable research platform must include both organic and inorganic constituents of bone ECM, such as CaP and collagen, to create complex bone cultures with properties similar to those observed in vivo.

Cell-derived ECM represents a promising alternative to tissue-derived ECM [16, 17], and its use in laboratory settings is simple, affordable, and free of ethical issues. Cell-derived ECM creates a microenvironment that mimics the in vivo condition, acting as a reservoir of cytokines and factors to facilitate cell growth and development [1, 18]. When preparing cell-derived ECM, researchers must address both the selection of an appropriate cell type for producing ECM and application of a suitable decellularization method. The osteosarcoma cell line Saos-2 and primary human osteoblasts have been shown to exhibit similar osteogenic differentiation capacities and generate comparable deposits of calcium in the same period [19]. The ECM derived from Saos-2 cells is mainly composed of collagen type I and type V and is mineralized by hydroxyapatite crystals [20]. These components of Saos-2 ECM are comparable to those of native bone ECM, making this cell line a good candidate for ECM production. Ammonium hydroxide (NH_4_OH) has been used as a decellularizing agent [21]; but the efficiency and applicability of NH_4_OH-mediated decellularization method require validation, as this decellularized Saos-2 ECM (DS-ECM) has only been used to quantify osteoclast activity rather than to support osteoprecursor cell growth and differentiation.

In the present study, we developed a decellularization method to generate DS-ECM as a platform for bone cell cultivation and differentiation. We then investigated the differentiation efficiency of a direct bone coculture system composed of mesenchymal stem cells (osteoblast progenitor cells) and monocytes (osteoclast progenitor cells) in DS-ECM and compared the results to those obtained from direct bone cocultures grown on an inorganic matrix coating of CaP or directly on conventional cell culture plastic (polystyrene).

## Materials and methods

### Chemicals and media

Chemicals were purchased from Sigma-Aldrich (St. Louis, MO, USA) or Carl Roth (Karlsruhe, Germany). Cell culture medium and supplements were purchased from Sigma-Aldrich or Gibco (Thermo Fisher Scientific, Waltham, MA, USA).

### Cell lines

Cells from the human osteoblast-like cell line Saos-2 (Deutsche Sammlung von Mikroorganismen und Zellkulturen [DSMZ], Leibniz, Germany) were used as osteogenic cells [22]. Saos-2 cells were cultured with RPMI 1640 medium supplemented with 5% v/v fetal bovine serum in a 5% CO_2_ incubator at 37 °C. The medium was replaced every 3–4 days.

Immortalized human mesenchymal stem cells (SCP-1 line, kindly provided by Dr. Matthias Schieker [23]) were used as osteoprogenitor cells. The cells were cultivated (37 °C, 5% CO_2_, and 100% humidity) in minimum essential medium Eagle α supplemented with 5% v/v fetal bovine serum. The cells were passaged at 80–90% confluence to avoid spontaneous differentiation. The medium was replaced every 3–4 days.

Cells from the human monocytic cell line THP-1 (DSMZ) were used as osteoclast precursor cells [22] and cultured in RPMI 1640 medium supplemented with 5% v/v fetal bovine serum. The medium was replaced every 3–4 days.

### Preparation of DS-ECM

#### Standard decellularization methods

Several decellularization procedures were adapted from previous publications [21, 24, 25] and tested. These standard protocols use chemicals (15 mM NH_4_OH, 0.5% v/v Triton X-100, and 1% w/v sodium dodecyl sulfate [SDS]) and cold or heat treatments. Table 1 summarizes the decellularization protocols. For ECM production, 1 × 10^4^ Saos-2 cells were seeded in 96-well plates (Greiner Bio-One, Kremsmünster, Austria) and cultured in osteogenic medium (RPMI 1640, 5% v/v fetal bovine serum, 200 μM L-ascorbic acid 2-phosphate, 5 mM β-glycerol phosphate, 25 mM HEPES, 1.5 mM CaCl_2_, and 5 μM cholecalciferol) for 13 days. To obtain cell-free matrices, the cells were incubated with 15 mM NH_4_OH with or without 1% w/v SDS or 0.5% v/v Triton X-100 for 30 min at room temperature. After incubation, the matrices were washed three times with phosphate-buffered saline (PBS). These matrices were then incubated at 4 °C, 37 °C, or 41 °C for 48 h and washed three times with PBS. To confirm that the DS-ECM was sterile, the matrices were incubated with culture medium for 2 days before cell seeding.

**Table 1:** Summary of the standard decellularization methods tested in this study

Protocol	Chemical treatment (30 min at room temperature)			Temperature (48 h)
	15 mM ammonium hydroxide	1% sodium dodecyl sulfate	0.5% Triton X-100	
1	+	−	−	4 °C
2	+	+	−	4 °C
3	+	−	+	4 °C
4	+	−	−	37 °C
5	+	+	−	37 °C
6	+	−	+	37 °C
7	+	−	−	41 °C
8	+	+	−	41 °C
9	+	−	+	41 °C

#### Modified decellularization methods

After 13 days of osteogenic culturing, the Saos-2 cells were treated with PBS or 15 mM NH_4_OH or 1% w/v SDS for 30 min. After incubation, the matrices were heat-treated at 41 °C, 43 °C, 45 °C, or 47 °C for 48 h. Table 2 lists the modified methods assessed in this paper. To confirm that the DS-ECM was sterile, the matrices were incubated with culture medium for 2 days before cell seeding.

**Table 2:** Summary of the modified decellularization methods tested in this study

Protocol	Chemical treatment (30 min at room temperature)			Temperature (48 h)
	Phosphate-buffered saline	15 mM ammonium hydroxide	1% sodium dodecyl sulfate	
A	+	−	−	41 °C
B	−	+	−	41 °C
C	−	−	+	41 °C
D	+	−	−	43 °C
E	−	+	−	43 °C
F	−	−	+	43 °C
G	+	−	−	45 °C
H	−	+	−	45 °C
I	−	−	+	45 °C
L	+	−	−	47 °C
M	−	+	−	47 °C
N	−	−	+	47 °C

### CaP coating

A synthetic inorganic matrix was coated with CaP under sterile conditions as described previously [12]. Two solutions, A and B, were freshly prepared and sterile-filtered before use (Table 3). Solution A (100 μL/well) was incubated (37 °C, 5% CO_2_, and 100% humidity) in 96-well plates for 3 days. After incubation, solution A was removed and solution B (100 μL/well) was added for 1 day (37 °C, 5% CO_2_, and 100% humidity). The wells were then washed three times with sterile water and air-dried overnight. The coated plates were incubated with culture medium for 2 days as a sterility control before cell seeding.

**Table 3:** Composition of the calcium phosphate coating

Substrate	Composition
Tris buffer	50 mM Tris base in demineralized water, pH 7.4
Calcium solution	25 mM calcium chloride, 1.37 M sodium chloride, and 15 mM magnesium chloride in Tris buffer, pH 7.4
Phosphate solution	11.1 mM disodium hydrogen phosphate and 42 mM sodium hydrogen carbonate in Tris buffer, pH 7.4
Solution A	50% Tris buffer, 25% calcium solution, and 25% phosphate solution
Solution B	2.25 mM disodium hydrogen phosphate, 4 mM calcium chloride, and 140 mM sodium chloride in Tris buffer with 4 M hydrogen chloride

### Cell seeding

SCP-1 (osteoprogenitor) cells and THP-1 (osteoclastic precursor) cells were used to generate the bone coculture system. THP-1 cells (2.4 × 10^4^ cells per well) were seeded in plates with and without DS-ECM or CaP coating in a culture medium supplemented with 200 nM phorbol 12-myristate 13-acetate to induce cell differentiation into adherent macrophages. After 24 h, SCP-1 cells (3 × 10^3^ cells per well) were seeded into the same well with osteogenic differentiation medium (50:50 mix of RPMI to minimum essential medium Eagle α, 2% v/v fetal bovine serum, 200 μM L-ascorbic acid 2-phosphate, 5 mM β-glycerol phosphate, 25 mM HEPES, 1.5 mM CaCl_2,_ and 5 μM cholecalciferol). No exogenous sources of macrophage colony-stimulating factor or RANKL (receptor activator of nuclear factor kappa-Β ligand) were added to induce osteoclastogenesis. The osteogenic differentiation medium was replaced twice a week.

### Resazurin conversion assay

Resazurin conversion assay (mitochondrial activity) was used to assess cell viability as described previously [26, 27]. Cells were washed once with PBS before addition of 100 μL of 0.0025% w/v resazurin in PBS. After 30 min of incubation at 37 °C, the concentration of the reaction product (resorufin) in each well was measured at 544 nm excitation and 590 nm emission wavelengths and corrected by subtracting the background values obtained from no-cell control wells.

### Cellular adenosine triphosphate (ATP) measurement

Cell viability was evaluated by measuring ATP content using a luminescence-based assay (CellTiter-Glo Luminescent Cell Viability Assay, Promega, Madison, WI, USA) by following the manufacturer’s protocol. The luminescence signal was measured using an Omega plate reader (BMG Labtech, Ortenberg, Germany) and corrected by subtracting the background values obtained from wells with assay solution with medium (without cells).

### Lactate dehydrogenase (LDH) release (extracellular)

The release of LDH into the supernatant is a marker of cell membrane damage [28]. The concentration of LDH in the medium was measured using a kit (CytoTox-ONE Homogeneous Membrane Integrity Assay; Promega). LDH reaction solution (100 µL) was added to each well, and the plates were incubated at room temperature for 10 min in the dark. Next, 50 µL of stop solution was added to obtain experimental LDH release. The cells were lysed using the cell lysis solution provided by the manufacturer to obtain maximum LDH release (positive control). The fluorescence of the osteogenic differentiation medium was used as the background value. The fluorescence signal was assessed using an Omega plate reader (BMG Labtech) and calculated as follows:

$${{{{{\mathrm{LDH}}}}}}\,{{{{{\mathrm{release}}}}}}\left[ \% \right] = \frac{{{{{{{\mathrm{Experimental}}}}}}\,{{{{{\mathrm{LDH}}}}}}\,{{{{{\mathrm{release}}}}}} - {{{{{\mathrm{Background}}}}}}}}{{{{{{{\mathrm{Total}}}}}}\,{{{{{\mathrm{LDH}}}}}}\,{{{{{\mathrm{release}}}}}} - {{{{{\mathrm{Background}}}}}}}} \times 100$$

### Calcein AM–Hoechst staining

Viable cells were visualized by calcein AM–Hoechst staining as described previously [27, 29]. The cells were washed once with PBS before addition of 100 µL of a staining solution (2 µM calcein-AM and 1 µM Hoechst 33342 in PBS) and incubation at 37 °C for 20 min. The cells were then washed with PBS and imaged with a fluorescence microscope (EVOS FL, Thermo Fisher Scientific, Carlsbad, CA, USA).

### Alkaline phosphatase activity

Alkaline phosphatase activity was determined as previously described [26, 27] by incubating cells with a reaction solution (0.2% w/v 4-nitrophenyl phosphate, 50 mM glycine, 1 mM MgCl_2_, and 100 mM Tris; pH 10.5). After a 30-min incubation, the concentration of the reaction product (4-nitrophenol) was quantified photometrically (λ = 405 nm; Omega plate reader, BMG Labtech).

### Tartrate-resistant acid phosphatase (TRAP) 5b activity

TRAP 5b activity as a marker of osteoclast activity was measured in culture supernatants as previously described [30, 31]. Culture supernatant (30 µL) was transferred to a new cell culture plate with 90 μL of TRAP 5b activity assay solution (0.2% w/v 4-nitrophenyl phosphate, 100 mM sodium acetate, and 50 mM sodium tartrate in demineralized water; pH 5.5) and incubated for 6 h at 37 °C. The reaction was stopped by adding 90 μL of 1 M NaOH, and the concentration of the reaction product (4-nitrophenol) was measured at 405 nm and corrected to background control (medium with assay solution). The results were normalized to mitochondrial activity (see 2.6 resazurin conversion assay).

### Carbonic anhydrase activity

The method of Bernhardt and colleagues [32] was used to measure carbonic anhydrase activity. Cells were incubated with carbonic anhydrase reaction solution (10 mM Tris at pH 7.5 and 60 mM sodium chloride with pH adjusted to 7.5, followed by addition of 200 mM 4-nitrophenyl acetate dissolved in ethanol), and the absorbance was measured every minute over a 15-min period by using an Omega plate reader (BMG Labtech). The rate of conversion of 4-nitrophenyl acetate to 4-nitrophenol was calculated and normalized to mitochondrial activity.

### Alizarin red staining

To assess matrix mineralization, cells were fixed with 100% ethanol overnight at −20 °C. The fixed cells were gently washed with tap water, stained with 50 µL of 0.5% w/v Alizarin Red solution (pH 4.0), and incubated at room temperature for 30 min. Excessive Alizarin Red was removed by washing the cells with tap water. Bright-field images of the stained cells were captured by microscopy (EVOS FL, Thermo Fisher Scientific). For quantitative measurement, the bound Alizarin Red was dissolved with 10% w/v cetylpyridinium chloride solution and quantified photometrically (λ = 562 nm; Omega plate reader) as described previously [31].

### Dot blot

Protein levels in cell culture supernatants were determined by dot blot as previously described [22]. Cell culture supernatants (40 µL) were applied to a nitrocellulose membrane by a dot blotter (Carl Roth). All transferred proteins were visualized by a solution containing 0.1% w/v Ponceau S in 1% v/v acetic acid. The membrane was blocked with 5% bovine serum albumin in Tris-buffered saline/Tween 20 (TBS-T; 10 mM Tris-HCl at pH 7.6, 0.15 mM NaCl, and 0.1% v/v Tween-20) at room temperature for 1 h. It was then incubated with anti-procollagen type I N-terminal propeptide (PINP; 1:1000 in TBS-T; Ref. abx131414, Abbexa, Cambridge, UK) and anti-osteonectin (1:1000 in TBS-T; Ref. sc-74295, Santa Cruz Biotechnology, Dallas, TX, USA) overnight at 4 °C. The following day, the membrane was incubated with the corresponding secondary antibodies (1:10,000 in TBS-T; Santa Cruz Biotechnology) for 2 h after washing. The protein signal was detected by a charge-coupled device camera (INTAS Science Imaging, Göttingen, Germany) after addition of an enhanced chemiluminescence substrate solution. The signal intensity was quantified using the ImageJ software (NIH, Bethesda, MD, USA).

### Statistical analysis

The results are presented as mean ± standard error of the mean (SEM). Each experiment was repeated twice for three biological replicates with at least three technical replicates (*n* ≥ 3). Group differences were analyzed by the nonparametric Mann–Whitney U test or Kruskal–Wallis H test, followed by Dunn’s multiple comparison test. The analysis was performed in GraphPad Prism (GraphPad Software 8.0, La Jolla, CA, USA).

## Results

### Saos-2 matrix formation over time

**Fig. 1:**
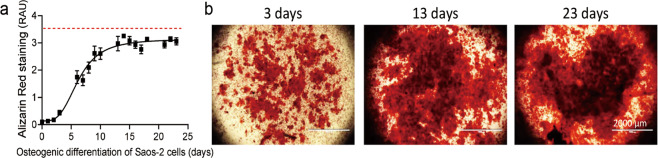
Matrix formation in Saos-2 cells. **a** Quantification of matrix deposition by Alizarin Red staining in relative absorbance units (RAU) at 560 nm. The red line shows the maximum absorbance detected at 560 nm (mean ± standard error of the mean; *N* = 4; *n* = 4). **b** Representative microscopy images of Alizarin Red staining. Scale bar: 2000 μm

Saos-2 cells were osteogenically differentiated for up to 23 days to determine the optimal time point for decellularization. The cells were fixed with ethanol at various time points to evaluate matrix formation by Alizarin Red staining. Calcium deposition in DS-ECM should be in the linear range and not reach saturation to detect later matrix formation and degradation from bone cells cultivated in DS-ECM. In the present study, calcium deposition increased linearly from day 4 until day 13 before reaching saturation between days 15 and 18 (Fig. 1a, b). Therefore, the DS-ECM from Saos-2 cells differentiated after 13 days was used to test different decellularization methods.

### NH_4_OH in combination with SDS at 41 °C exhibits the highest degree of Saos-2 decellularization among standard decellularization methods

**Fig. 2:**
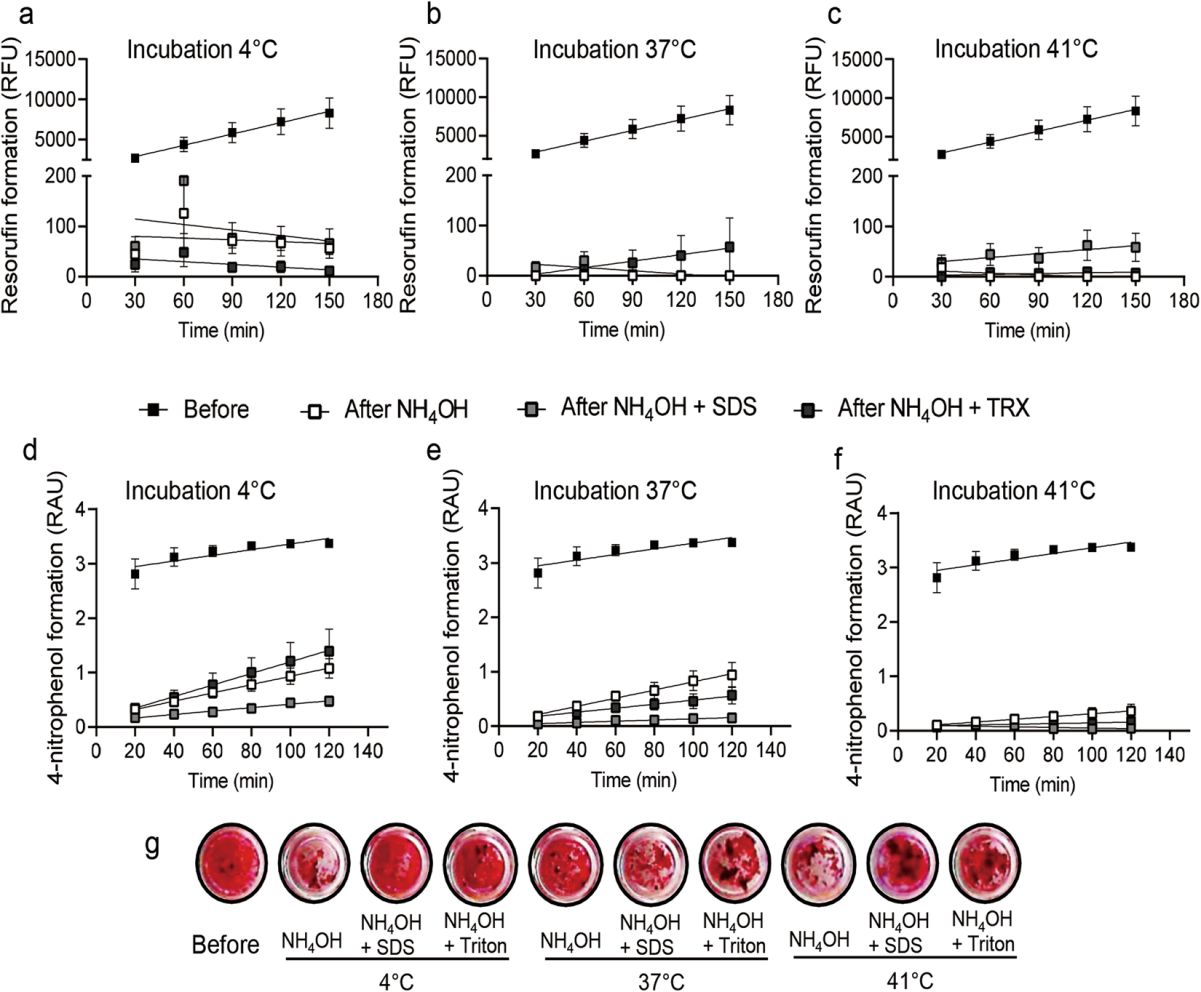
Decellularization efficiency of standard decellularization treatments. Resorufin formation presented in relative fluorescence units (RFU) before and after treatment with the chemical decellularization agents ammonium hydroxide (NH_4_OH), sodium dodecyl sulfate (SDS), and Triton X-100 (TRX) in various combinations as well as with protein heat inactivation at (**a**) 4 °C, (**b**) 37 °C, and (**c**) 41 °C. Alkaline phosphatase activity is represented by 4-nitrophenol formation in relative absorbance units (RAU) at 405 nm at several time points before and after treatment with chemical decellularization agents and protein heat inactivation at (**d**) 4 °C, (**e**) 37 °C, and (**f**) 41 °C. **g** Representative images of Alizarin Red-stained cells before and after treatment with standard decellularization agents. Results represent the mean ± standard error of the mean (*N* = 3; *n* = 3)

To compare the efficiency of standard decellularization methods, we analyzed mitochondrial activity, alkaline phosphatase activity, and Alizarin Red staining in Saos-2 cells. Measurement of resazurin conversion showed that, before decellularization, the fluorescence intensity of resorufin formation in Saos-2 cells increased linearly over time and, at 150 min, reached an intensity approximately twofold higher than the basal level of intensity at 30 min (Fig. 2a–c). None of the nine decellularization treatments caused a gradual increase in resorufin formation in exposed cells (Fig. 2a–c). Measurement of alkaline phosphatase activity showed that, before decellularization, 4-nitrophenol formation in Saos-2 cells linearly increased over time. Cells decellularized by cold treatment at 4 °C with or without one of the three chemical methods exhibited a linear increase in 4-nitrophenol formation over time (Fig. 2d). In Saos-2 cells exposed at 37 °C to NH_4_OH combined with SDS, the rate of 4-nitrophenol formation remained unchanged for up to 120 min (Fig. 2e). A similar trend was observed in Saos-2 cells exposed at 41 °C to NH_4_OH combined with SDS or Triton X-100. The smallest slope of alkaline phosphatase activity was observed in cells exposed at 41 °C to NH_4_OH combined with SDS (Fig. 2f). Alizarin Red staining showed that calcium deposition was strongly affected in Saos-2 cells exposed at 37 °C to NH_4_OH combined with SDS (Fig. 2g and Figure S1). In contrast, calcium deposition was largely unaffected in Saos-2 cells decellularized with NH_4_OH combined with SDS or Triton X-100 at 41 °C. (Fig. 2g and Fig. S1). Overall, combined treatment with NH_4_OH and SDS at 41 °C produced the highest degree of Saos-2 decellularization and did not affected calcium deposition. We used this procedure to investigate cell repopulation attachment on the DS-ECM.

### DS-ECM decellularized by a combination of NH_4_OH and SDS at 41 °C is cytotoxic

**Fig. 3:**
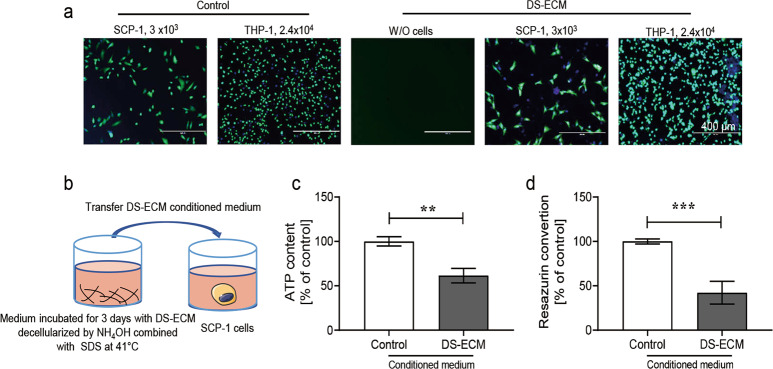
Cell attachment and viability on a matrix decellularized by ammonium hydroxide (NH_4_OH) in combination with sodium dodecyl sulfate (SDS) at 41 °C. **a** SCP-1 cells and THP-1 cells were seeded on a Saos-2 matrix decellularized by combined treatment with NH_4_OH and SDS at 41 °C. The same numbers of SCP-1 and THP-1 cells were also seeded in untreated wells as controls. After 24 h, live cells were visualized by calcein-AM (green) and Hoechst (blue) staining. Scale bar: 400 µm. **b** SCP-1 cells were treated with medium incubated with the decellularized Saos-2 extracellular matrix (DS-ECM) for 3 days. **c** Cellular adenosine triphosphate (ATP) content and (**d**) mitochondrial activity were used as markers of cell viability. Statistical significance was determined by the nonparametric Mann–Whitney U test. Results represent the mean ± standard error of the mean. **p *<* 0.01, ***p *<* 0.001 (*N* = 3; *n* = 3). W/O, without

Decellularization by treatment with a combination of NH_4_OH and SDS at 41 °C produced the highest degree of Saos-2 cell detachment and did not affect calcium deposition. Next, we performed calcein-AM staining to assess cell (osteoprogenitor cells) attachment and repopulation on the DS-ECM. SCP-1 cells (osteogenic precursor cells; 3 × 10^3^ cells per well) and THP-1 cells (osteoclastic precursor cells; 2.4 × 10^4^ per well) were seeded on the DS-ECM. The same numbers of bone cells were also seeded separately in untreated wells (in polystyrene cell culture plate) as controls. After 24 h of culture, viable cells growing in untreated wells and on the DS-ECM were stained with calcein AM and visualized (Fig. 3a). Although the density of THP-1 cells in the DS-ECM and control groups was comparable, that of SCP-1 cells was slightly lower on DS-ECM than on the plastic plate, suggesting that DS-ECM produced by combined treatment with NH_4_OH and SDS at 41 °C might be toxic to SCP-1 cells.

To further study the toxicity of the DS-ECM or compounds remaining from the decellularization process in SCP-1 cells, the DS-ECM was cultured in osteogenic differentiation medium for 3 days. The conditioned DS-ECM medium was collected and centrifuged (600 × *g* for 10 min) to remove cell debris. Then, the clear supernatant was added to SCP-1 cells cultured in a fresh plastic plate (3 × 10^3^ cells per well; Fig. 3b). Osteogenic differentiation medium incubated in untreated wells (without DS-ECM) was used as a control. After 48 h of incubation, the ATP content and mitochondrial activity in SCP-1 cells exposed to the conditioned DS-ECM medium had decreased by ~38% and 57%, respectively (Fig. 3c and d). These findings demonstrated that the decellularization method involving NH_4_OH and SDS at 41 °C was toxic to SCP-1 cells and required modification.

### Heat treatment results in better decellularization at 47 °C than at lower temperatures

To avoid the cytotoxicity associated with SDS, we tested additional decellularization methods (Section 2.3.2). These methods combined the use of chemicals (15 mM NH_4_OH and 1% w/v SDS) with heat treatment (41 °C, 43 °C, 45 °C, and 47 °C). Heat treatment was also performed in the presence of PBS to investigate the decellularization efficiency of heat in the absence of chemicals. Mitochondrial activity, alkaline phosphatase activity, and Alizarin Red staining were assessed to determine the efficiency of the decellularization process.

**Fig. 4:**
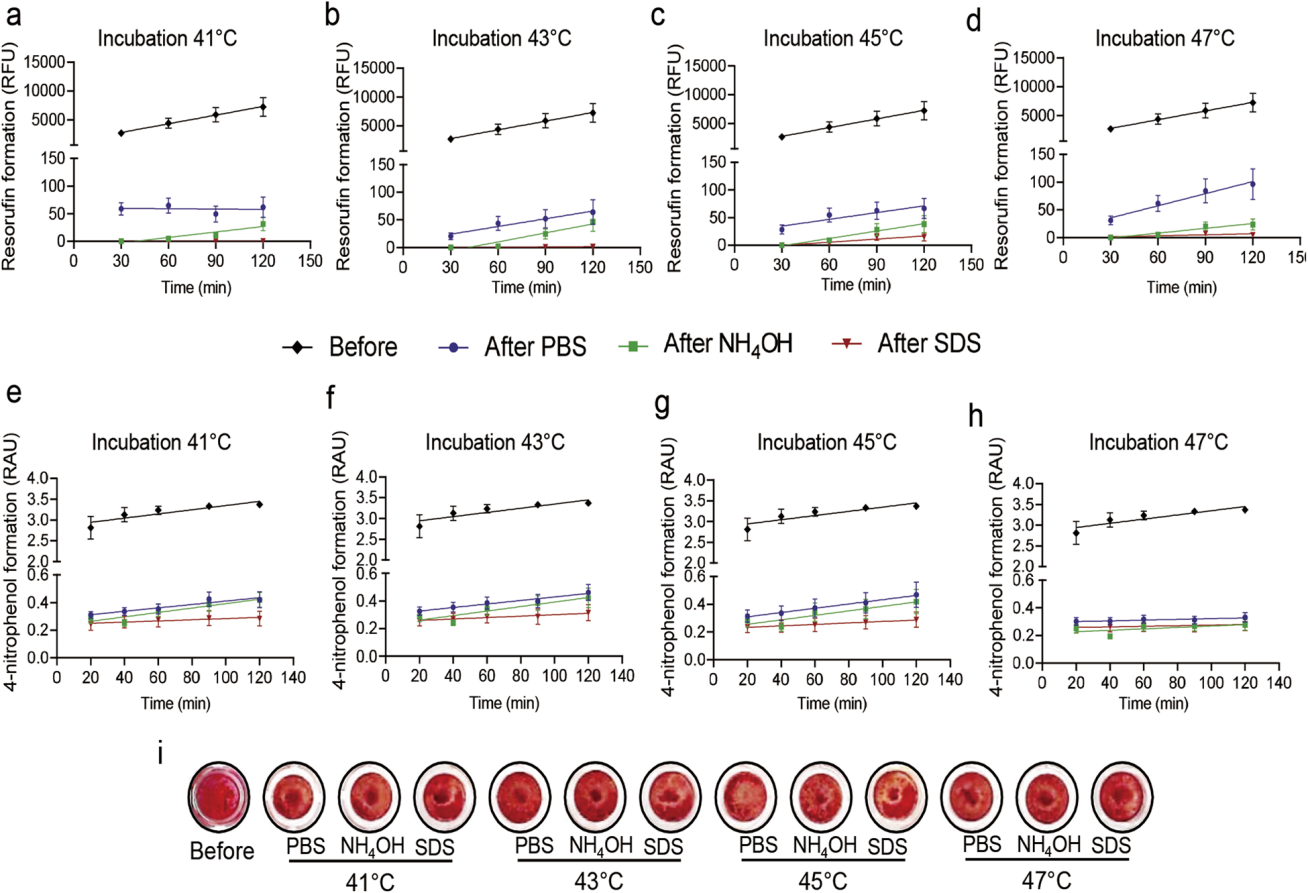
Decellularization efficiency of modified standard treatments. Resorufin formation represented in relative fluorescence units (RFU) of intensity before and after decellularization with phosphate-buffered saline (PBS), ammonium hydroxide (NH_4_OH), and sodium dodecyl sulfate (SDS) combined with heat inactivation at (**a**) 41 °C, (**b**) 43 °C, (**c**) 45 °C, and (**d**) 47 °C. 4-Nitrophenol formation represented in relative absorbance units (RAU) of intensity at 405 nm before and after treatment with modified chemical decellularization methods and protein heat inactivation at (**e**) 41 °C, (**f**) 43 °C, (**g**) 45 °C, and (**h**) 47 °C. **i** Representative images of Alizarin Red-stained cells before and after treatment with modified decellularization methods. Results represent the mean ± standard error of the mean (*N* = 3; *n* = 3)

Measurement of resazurin conversion showed no increase in resorufin formation in Saos-2 cells over time for any of the decellularization methods tested (Fig. 4a–d). In line with the findings described above (Section 3.2), residual alkaline phosphatase activity was detectable at 41 °C (compare Fig. 2d–f with Fig. 4e–h). Alkaline phosphatase activity was low after NH_4_OH treatment and undetectable after SDS treatment combined with heat treatment between 41 °C and 45 °C (Fig. 4e–g). Alkaline phosphatase activity was also low when the Saos-2 cells were heated between 41 and 45 °C without chemical treatment, i.e., when PBS was used. Incubation at 47 °C resulted in no detectable alkaline phosphatase activity in any of the decellularization methods tested, even when no chemical agent was used (Fig. 4h). Alizarin Red staining showed that calcium deposition was unaffected in Saos-2 cells exposed to various modified decellularization methods (Fig. 4i and Figure S2).

### The highest degree of Saos-2 decellularization without cytotoxicity is achieved by 47 °C treatment alone

**Fig. 5:**
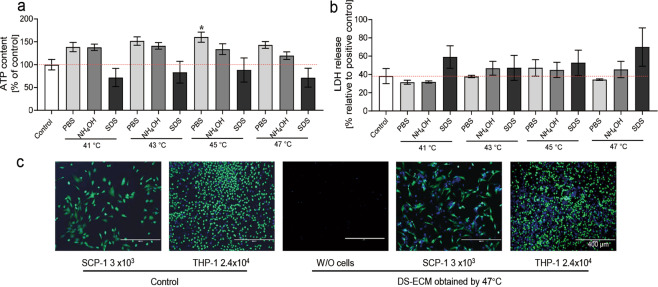
Cell growth on the decellularized Saos-2 extracellular matrix (DS-ECM) obtained by modified decellularization treatments. After 48 h of incubation, adenosine triphosphate (ATP) release (**a**) and lactate dehydrogenase (LDH) content (**b**) were measured in SCP-1 cells. **c** SCP-1 cells and THP-1 cells were seeded on a Saos-2 matrix decellularized by heat inactivation at 47 °C. The same numbers of cells were also seeded in untreated wells as controls. After 24 h, live cells were visualized by calcein-AM dye (green) and Hoechst staining (blue). Scale bar: 400 µm. Results represent the mean ± standard error of the mean. The values were compared by nonparametric one-way analysis of variance. *p < 0.05 (*N* = 3; *n* = 2). NH_4_OH, ammonium hydroxide; SDS, sodium dodecyl sulfate; W/O, without

To evaluate the cytotoxicity of the DS-ECM generated by the modified decellularization methods (Section 2.3.2), the DS-ECM was incubated with osteogenic differentiation medium, and the DS-ECM-conditioned medium was added to SCP-1 cell cultures. Osteogenic differentiation medium incubated in untreated wells was used as a control. The ATP content in SCP-1 cells treated for 48 h with the conditioned medium increased after heat inactivation at 41 °C, 43 °C, 45 °C, and 47 °C concomitantly with PBS or NH_4_OH. However, conditioned medium from the SDS-treated group caused a decrease in ATP levels by 28, 17, 12, and 29%, respectively (Fig. 5a).

Greater LDH release, indicating increased cellular damage, was observed in SCP-1 cells exposed to medium conditioned with matrices decellularized by SDS and heat treatment (any temperature; Fig. 5b). The lowest LDH release was measured in SCP-1 cells treated with medium conditioned with decellularized matrices obtained after heat (any temperature) and PBS treatment. These results suggest that the DS-ECM might still contain residual SDS after decellularization and that this residue is toxic to SCP-1 cells. In summary, heat treatment at 47 °C produced optimal Saos-2 matrix decellularization and no cytotoxicity.

To evaluate cell attachment to the DS-ECM obtained by heat treatment at 47 °C, 3 × 10^3^ SCP-1 cells or 2.4 × 10^4^ THP-1 cells were seeded on DS-ECM. The same cell numbers were also seeded in untreated wells and used as controls. Calcein AM and Hoechst 33342 staining (Fig. 5c) showed that viable SCP-1 and THP-1 cells were attached to the DS-ECM with a density comparable to that of the control group.

### DS-ECM supports osteoclast differentiation and matrix mineralization more efficiently than CaP coating

**Fig. 6:**
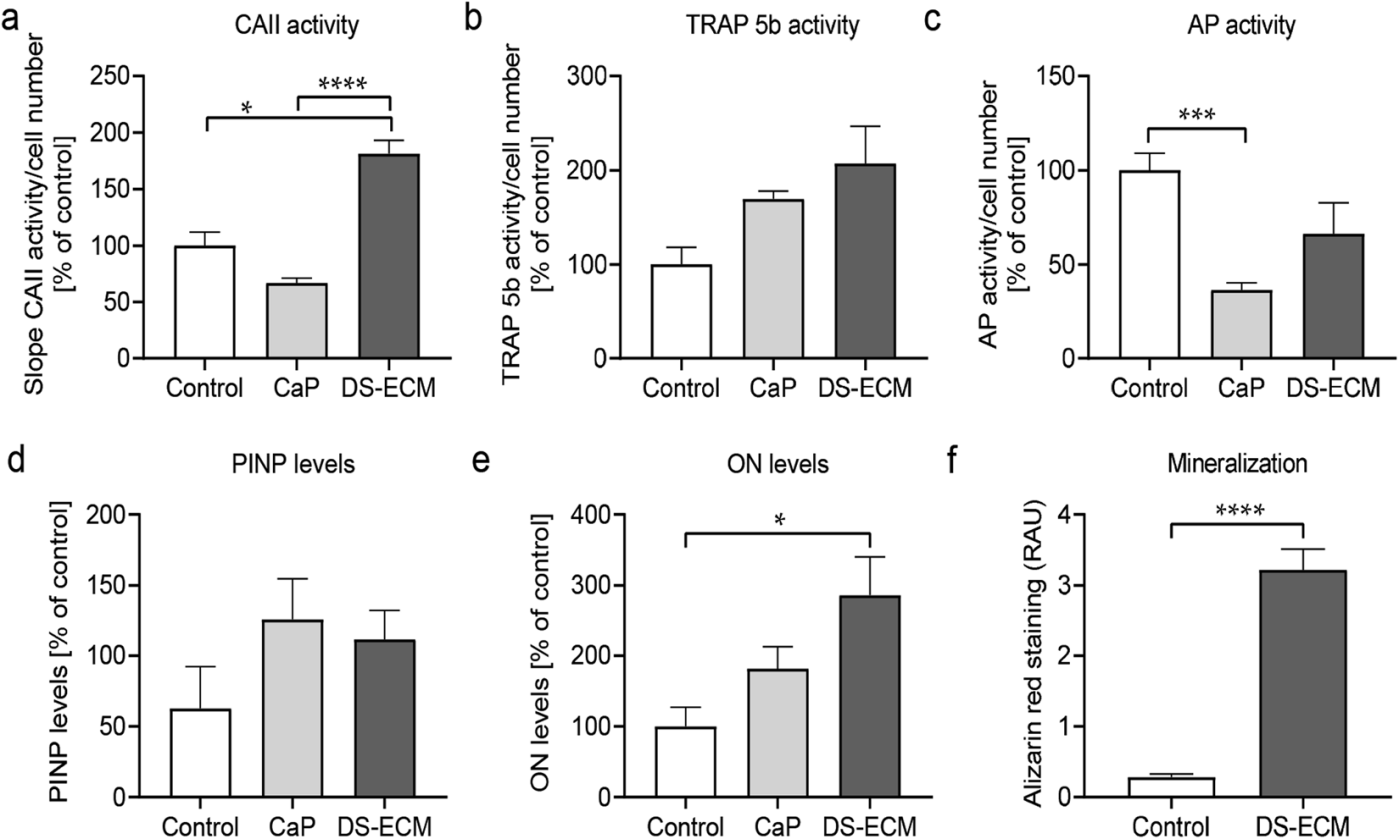
Cell function in the bone coculture system cultivated on various culture platforms. The SCP-1/THP-1 cell bone coculture system was seeded on plastic plates (control), calcium phosphate (CaP)-coated surface, or decellularized Saos-2 extracellular matrix (DS-ECM). On day 7, osteoclast function was determined by carbonic anhydrase (CA II) activity (**a**) and tartrate-resistant acid phosphatase 5b (TRAP 5b) activity (**b**). **c** Osteoblast function was determined by alkaline phosphatase (AP) activity. On day 14, dot blot analysis of culture supernatants was performed to determine the relative levels of (**d**) procollagen type I N-terminal propeptide (PINP) and (**e**) osteonectin (ON). **f** Mineralization was measured by Alizarin Red staining on day 14 and corrected by subtracting the background levels. The values were compared by a nonparametric Mann–Whitney U test or nonparametric one-way analysis of variance. Results represent mean ± standard error of the mean. *p < 0.05; ***p < 0.001; ****p < 0.0001 (*N* = 3; *n* = 2–3). RAU, relative absorbance units

To compare the osteoblastic and osteoclastic functions of bone cells cultured on DS-ECM, SCP-1 and THP-1 cells were seeded on CaP-coated and untreated (controls) surfaces. Bone cocultures grown on the CaP-coated and untreated surfaces showed comparable mitochondrial activity after 7 days of culture (Figure S3). Osteoclast activity was evaluated by measuring TRAP 5b and carbonic anhydrase activity after 7 days of culturing. Carbonic anhydrase activity was significantly higher in cells cultured on the DS-ECM surface than in cells cultured on the CaP-coated or untreated surface (Fig. 6a). TRAP 5b activity in THP-1 cells was twice as high on DS-ECM than on the untreated surface, although the difference was not statistically significant (Fig. 6b). The coculture system exhibited higher TRAP 5b activity on DS-ECM than on CaP coating (206% vs. 169%; *p* > 0.05). Alkaline phosphatase activity, an early marker of osteogenic differentiation [27], was also measured after 7 days in culture; it was significantly lower (~60%) in the coculture system cultured on the CaP-coated surface than in that cultured on the untreated surface. The alkaline phosphatase activity in cells cultured on DS-ECM was 30% lower than the activity in cells cultured on the untreated surface Fig. 6c). After 14 days of differentiation, the supernatants of bone cells cultured on the three surfaces were analyzed for two matrix remodeling markers, PINP, a bone formation marker [33], and osteonectin, a late osteogenic differentiation marker [34]. PINP levels were comparable between the supernatants from cells cultured on DS-ECM and CaP coating (Fig. 6d). Interestingly, the highest levels of osteonectin were measured in the supernatant of cells cultured on DS-ECM (2.85-fold greater than that in the control), followed by cells cultured on a CaP-coated surface (1.81-fold greater than control levels; Fig. 6e). Matrix mineralization, a marker of functionality and differentiation in osteoblasts [31], was assessed by Alizarin Red staining after 14 days of culture (Fig. 6f). The DS-ECM significantly increased calcium deposition by the coculture, when compared with the deposition from cells cultured on the untreated surface (Fig. 6f). Cells cultured on the CaP-coated surface could not be analyzed by Alizarin Red staining because of the acidic conditions of the staining solution (pH 4.0), which caused detachment of the CaP coating.

## Discussion

In the present study, we developed a procedure for generating a decellularized ECM from a human cell line. The ECM was further used for bone cell cultivation and differentiation, and it could better mimic the bone microenvironment in vivo. We used the human osteogenic cell line Saos-2 to produce an ECM that includes organic and inorganic constituents, as experiments conducted on a plastic plate or by using single ECM constituents have demonstrated that these surfaces are not optimal for culturing bone cells [35, 36]. Saos-2 cells are known to produce and deposit large quantities of calcium, comparable to the deposits from human osteoblasts, and to exhibit enhanced alkaline phosphatase activity during osteogenic differentiation [37, 38]. In our study, calcium deposition by Saos-2 cells reached saturation after 15–18 days of differentiation, indicating that 13 days is the cultivation period that yields optimal matrix deposition in this cell line.

An ideal decellularization method should remove cell constituents and eliminate enzymatic activity associated with matrix deposition while maintaining the structure of the ECM. The potential toxicity of residual chemicals used for decellularization procedures (e.g., SDS and NH_4_OH) is a considerable limitation. NH_4_OH (15 mM) combined with treatment at 4 °C has been reported to decellularize a matrix of Saos-2 cells [21]. In our study, this protocol indeed successfully decellularized a matrix of Saos-2 cells; however, we observed residual alkaline phosphatase activity. Because of this, we compared the decellularization efficiency of standard protocols by combining two chemical and temperature-specific treatments. The non-ionic Triton X-100 and ionic SDS detergents are potent decellularization agents with protein denaturation properties [24, 25]. Therefore, we used Triton X-100 and SDS to enhance decellularization and reduce residual enzymatic activity. We found that alkaline phosphatase activity was inhibited more effectively when NH_4_OH was combined with SDS or Triton X-100 rather than when used alone. The highest degree of decellularization by these standard methods was achieved by a combination of NH_4_OH and SDS at 41 °C. However, the DS-ECM obtained from this method was cytotoxic to SCP-1 and THP-1 cells, and this cytotoxicity was presumably attributable to the residual SDS in the matrix, as previously demonstrated by Rieder and colleagues [39]. Because we observed that heat treatment at 47 °C without chemicals was sufficient to yield a DS-ECM without residual alkaline phosphatase activity or cytotoxicity, we elected to continue with this approach and evaluate whether this matrix could improve bone cell characteristics.

When assessing the effects of DS-ECM on bone cell function, both bone formation and bone resorption should be investigated. Our previous study showed that osteogenic precursor SCP-1 cells release macrophage colony-stimulating factor and RANKL, which enhance osteoclast differentiation in THP-1 cells [19]. For this reason, the SCP-1/THP-1 coculture in the present study displayed features of osteogenic differentiation.In the present study, SCP-1/THP-1 cell function was determined in cocultures grown on DS-ECM, on a CaP-coated surface, or directly in untreated polystyrene cell culture plates. SCP-1/THP-1 coculturing on DS-ECM resulted in higher activity of carbonic anhydrase and TRAP 5b, functional markers of osteoclast [31, 40]), than coculturing on a CaP-coated or conventional plastic surface. A prior study highlighted the importance of cell culture surfaces in osteoclast fusion and functionality [15]. For instance, only the presence of both organic and inorganic bone matrix constituents promotes the expression of annexin A8, which is required for fusion of mononuclear cells to generate multinucleated osteoclasts. This may explain why osteoclast differentiation was better supported by DS-ECM than by CaP coating in our study.

Osteogenic differentiation of mesenchymal stem cells is a complex process that includes proliferation, matrix maturation, and mineralization [26, 27]. Several proteins are involved in osteogenic differentiation and exert their effects at different stages of the maturation process. For example, alkaline phosphatase is expressed during the early phases of osteogenic differentiation [27]. PINP is secreted by osteoblasts during collagen formation [33]. Late osteogenic differentiation is related to matrix mineralization. Osteonectin, secreted by mature osteoblasts, also affects matrix mineralization by linking mineral crystals to collagen [34, 41]. In the present study, relative to conventional plastic well plates, both DS-ECM and CaP coating reduced the activity of alkaline phosphatase in the SCP-1/THP-1 coculture, and this effect may be associated with the development and maturation of osteoblast precursor cells. This is in accordance with the findings of a previous study, where mesenchymal stem cells showed lower alkaline phosphatase activity at a later stage of osteogenic differentiation than at an early stage [42]. In addition, the higher levels of osteonectin observed in SCP-1/THP-1 cocultures on DS-ECM and CaP-coated surfaces than on conventional plastic plates support the enhanced osteogenic differentiation observed in our study. Although PINP levels did not differ between cells cultured on DS-ECM and those cultured on CaP-coated surfaces, the higher levels of osteonectin measured in the supernatant of the former indicate that DS-ECM can promote matrix mineralization better than CaP coating. Furthermore, culturing on DS-ECM yielded significantly higher calcium deposition than culturing on conventional cell culture plates.

From our findings, we believe that the DS-ECM developed here represents a simple-to-use and cost-efficient platform for cultivating and differentiating an SCP-1/THP-1 coculture.

## Conclusion

We have developed a simple and efficient method of generating a decellularized ECM from the human osteogenic cell line Saos-2. Treatment at 47 °C can effectively decellularize a matrix while preserving calcium deposition. The biocompatibility of the DS-ECM was confirmed by the successful attachment and survival of bone cells. In addition, DS-ECM enhanced osteoprogenitor cell function and differentiation. DS-ECM obtained by treatment at 47 °C provides a promising platform for differentiating bone cells, which can be used to study the effects of drugs on bone cell homeostasis, molecular pathways associated with metabolic bone disorders, and biological processes occurring in human bones.

## Supplementary information

Supplementary Figure S1

Supplementary Figure S2

Supplementary Figure S3

## Data Availability

The datasets and/or analysed generated during the current study are available from the corresponding author on reasonable request.
