# Supplementary figures and images for: A simple method for decellularizing a cell-derived matrix for bone cell cultivation and differentiation
Source: J Mater Sci Mater Med. 2021 Sep 15;32(9):124. doi: 10.1007/s10856-021-06601-y (PMC8443471; doi:10.1007/s10856-021-06601-y)

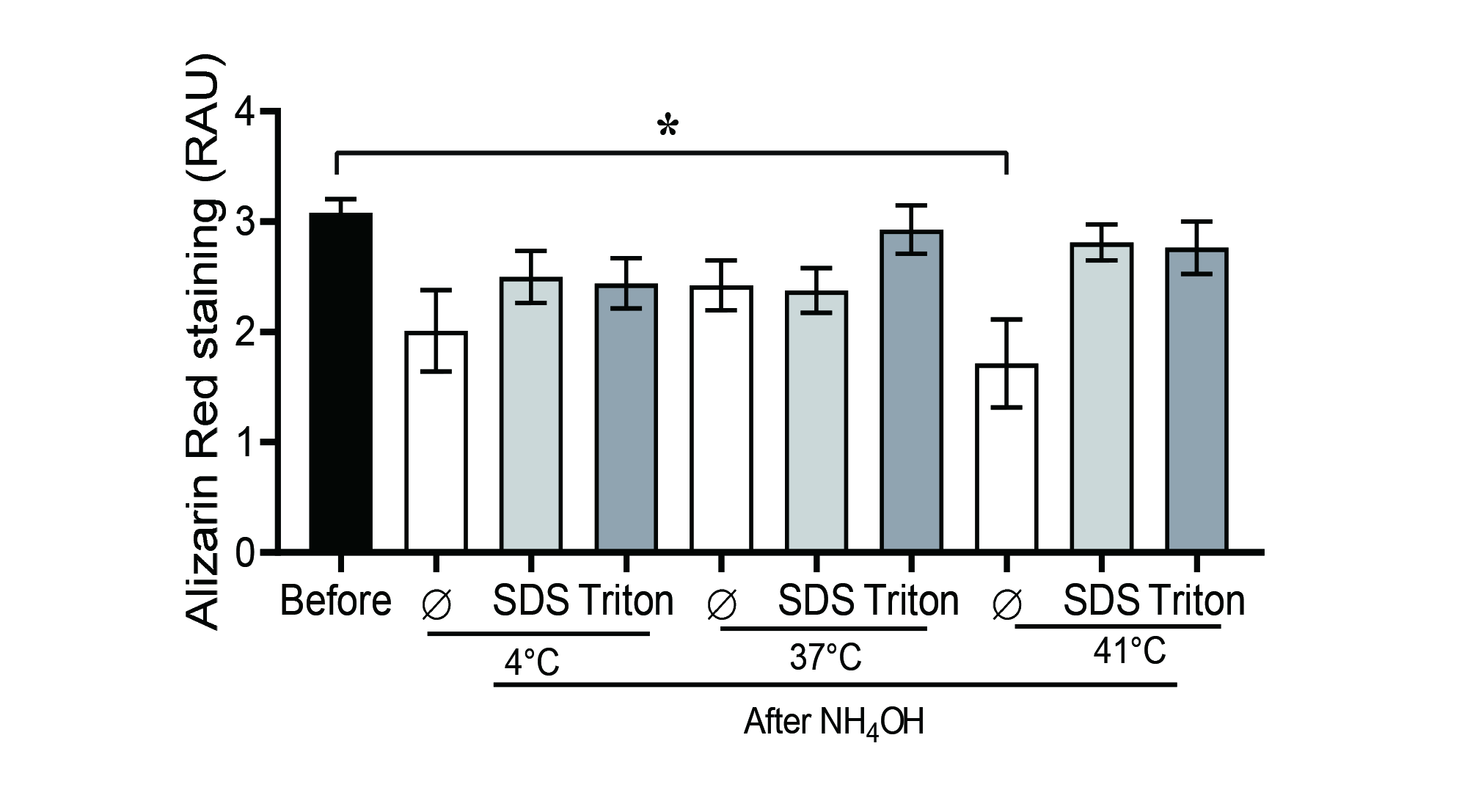

Supplement: Supplementary file 1 — Supplementary Figure S1 [file 10856_2021_6601_MOESM1_ESM.tif]

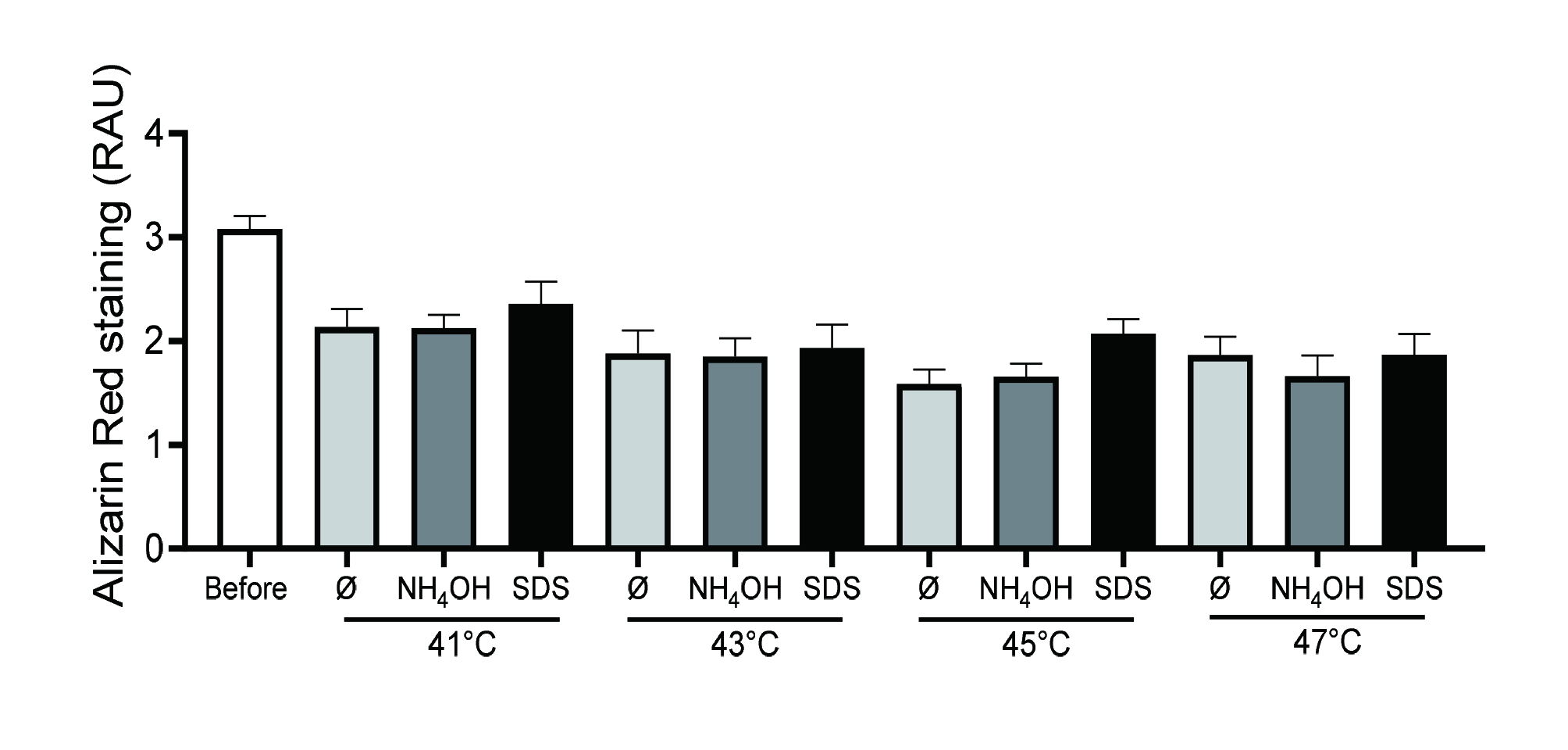

Supplement: Supplementary file 2 — Supplementary Figure S2 [file 10856_2021_6601_MOESM2_ESM.tif]

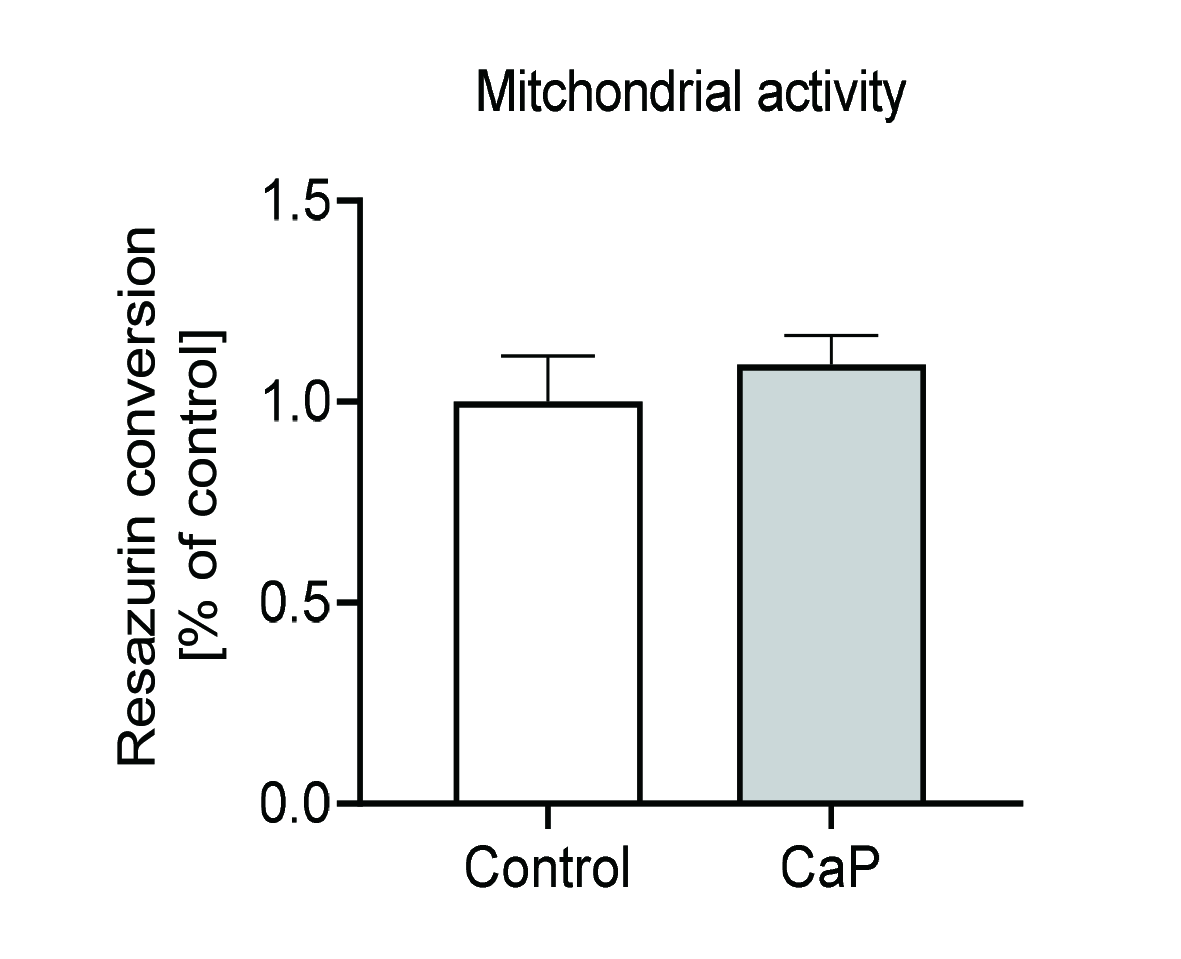

Supplement: Supplementary file 3 — Supplementary Figure S3 [file 10856_2021_6601_MOESM3_ESM.tif]
